# Supplementary material for: SERPINB3 (SCCA1) inhibits cathepsin L and lysoptosis, protecting cervical cancer cells from chemoradiation
Source: Commun Biol. 2022 Jan 12;5:46. doi: 10.1038/s42003-021-02893-6 (PMC8755728; doi:10.1038/s42003-021-02893-6)
Supplement: Supplementary file 2 — Supplementary Information [file 42003_2021_2893_MOESM2_ESM.pdf]

Supplementary Information

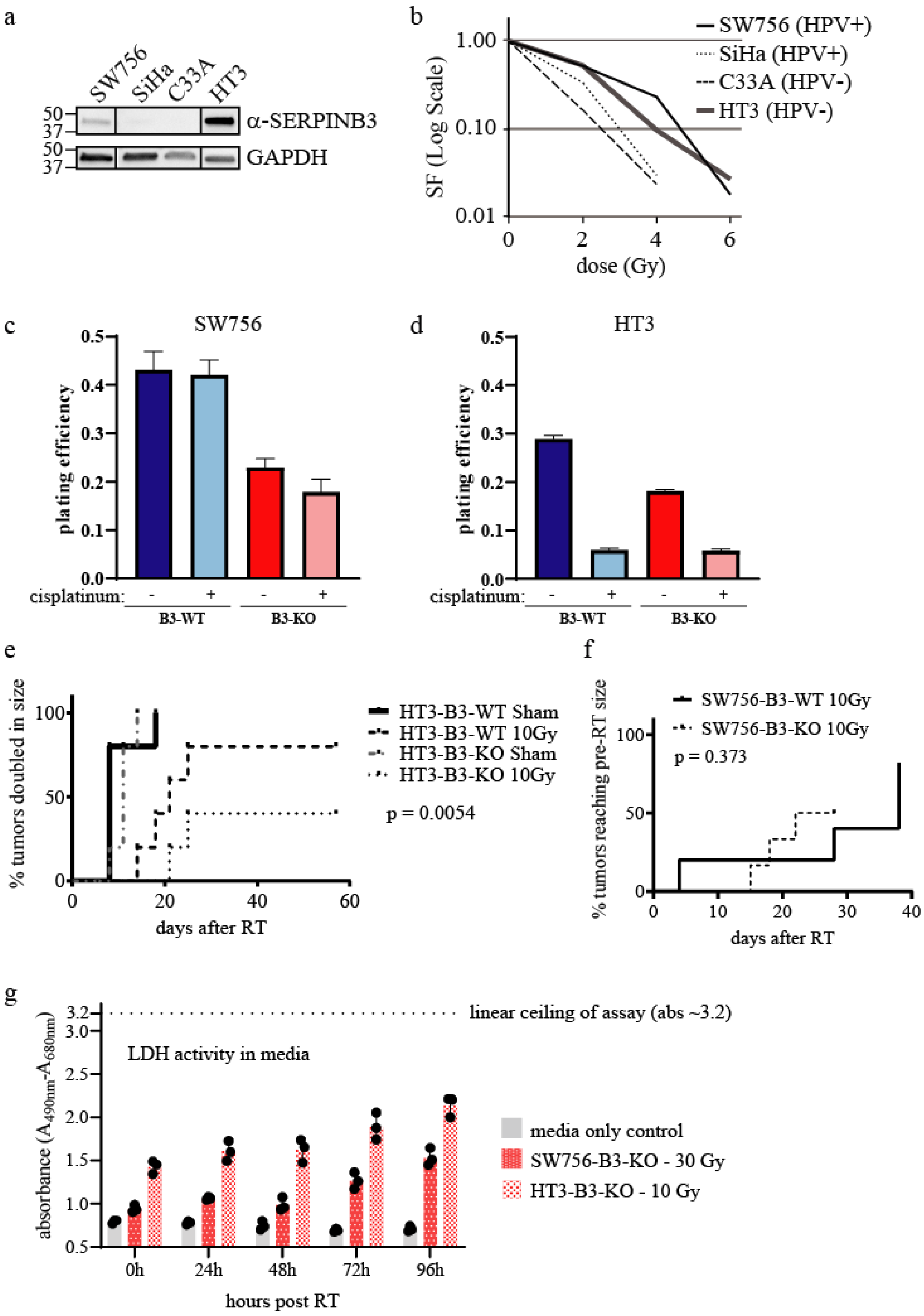

Supplemental Figure 1. a) Western blot showing steady state total cell lysate SERPINB3 protein levels in untreated SW756, SiHa, C33A and HT3 cells. Vertical black bars indicate spliced lanes on the same blot. **b)** Clonogenic survival of the four cell lines, two HPV+ and two HPV-). **c)** Plating efficiency (PE) of SW756-B3-WT and -B3-KO lines treated with vehicle control (-) or cisplatinum (+). **d)** PE of HT3-B3-WT and -B3-KO lines treated with vehicle control or cisplatin. Triplicate wells of one biologic replicate is shown for (c) and (d). Three biologic replicates were performed for each experiment. e) Kaplan-Meier curves of percent of HT3 tumors that have doubled in size with log-rank (Mantel-Cox) test p-value shown. f) Kaplan-Meier curves of percent of irradiated SW756 tumors reaching pre-RT size over time. P-value for log-rank statistic is shown. One experiment is shown with 5 mice per group. g) Lactate dehydrogenase (LDH) enzyme activity in the media of cells at the indicated times after treatment with 10 Gy (HT3-B3-KO) or 30 Gy (SW756-B3-KO) radiation. Control media only was included and cell titration with cell lysis (not shown) was used to determine the ceiling of the linear threshold of the assay as indicated. Individual data points represent triplicate wells of a representative one of three biologic replicate experiments performed.

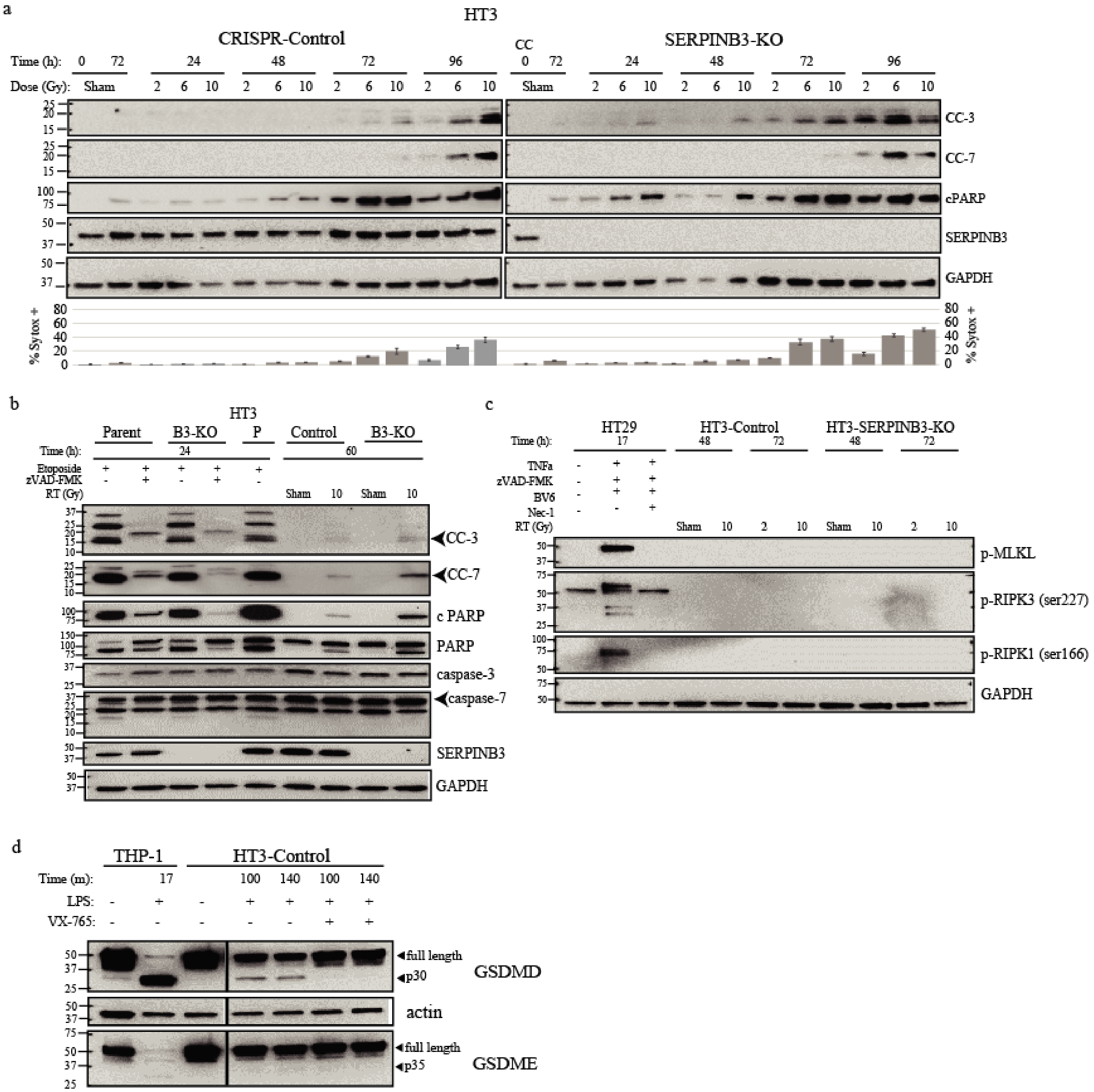

Supplemental Figure 2. **a)** Full Western blot of all dose levels and time points abbreviated in Figure 4A. % Sytox positive cells for each condition is shown beneath the Western blot. **b)** Control Western blot for markers of apoptosis, cleaved caspase-3 (CC-3), cleaved caspase-7 (CC-7) and PARP cleavage (cPARP). HT3 cells treated with etoposide to induce apoptosis, with or without zVAD-FMK pre-treatment to inhibit pan-caspase activity. Sham and 10Gy RT samples from HT3-C and –B3-KO cells are shown for reference. **c)** Control Western blot for markers of necroptosis, p-RIPK3, p-RIPK1 and p-MLKL. HT29 cells were treated with TNFα + zVAD-FMK + SMAC mimetic BV6 to induce necroptosis with or

without necrostatin-1 (Nec-1). Sham and radiated HT3-C and –B3-KO cells are shown as reference. **d)** Control Western blot for markers of pyroptosis, GSDMD cleavage. THP-1 cells or HT3-C cells were treated with LPS for the indicated times to induce pyroptosis, with or without caspase-1 inhibitor VX-765.

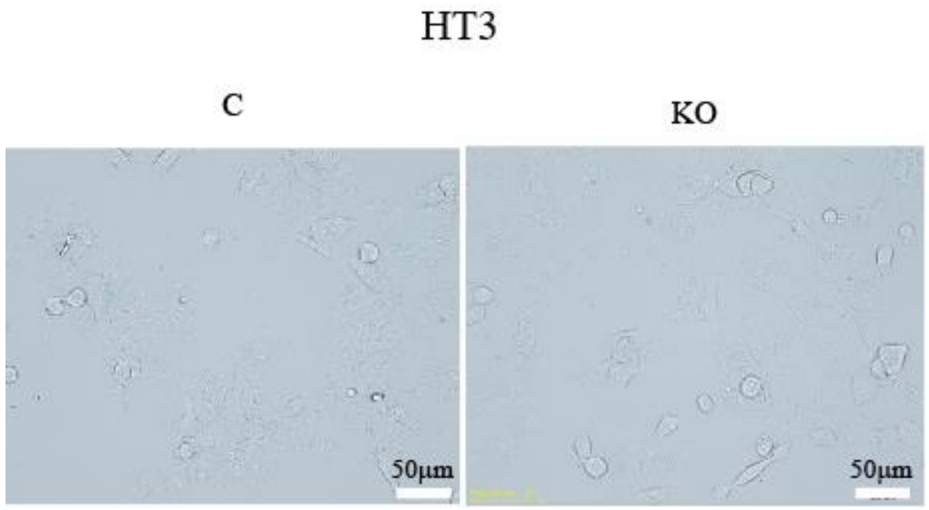

Supplemental Figure 3. Representative light microscope image of SA-βgal staining of HT3 cells.

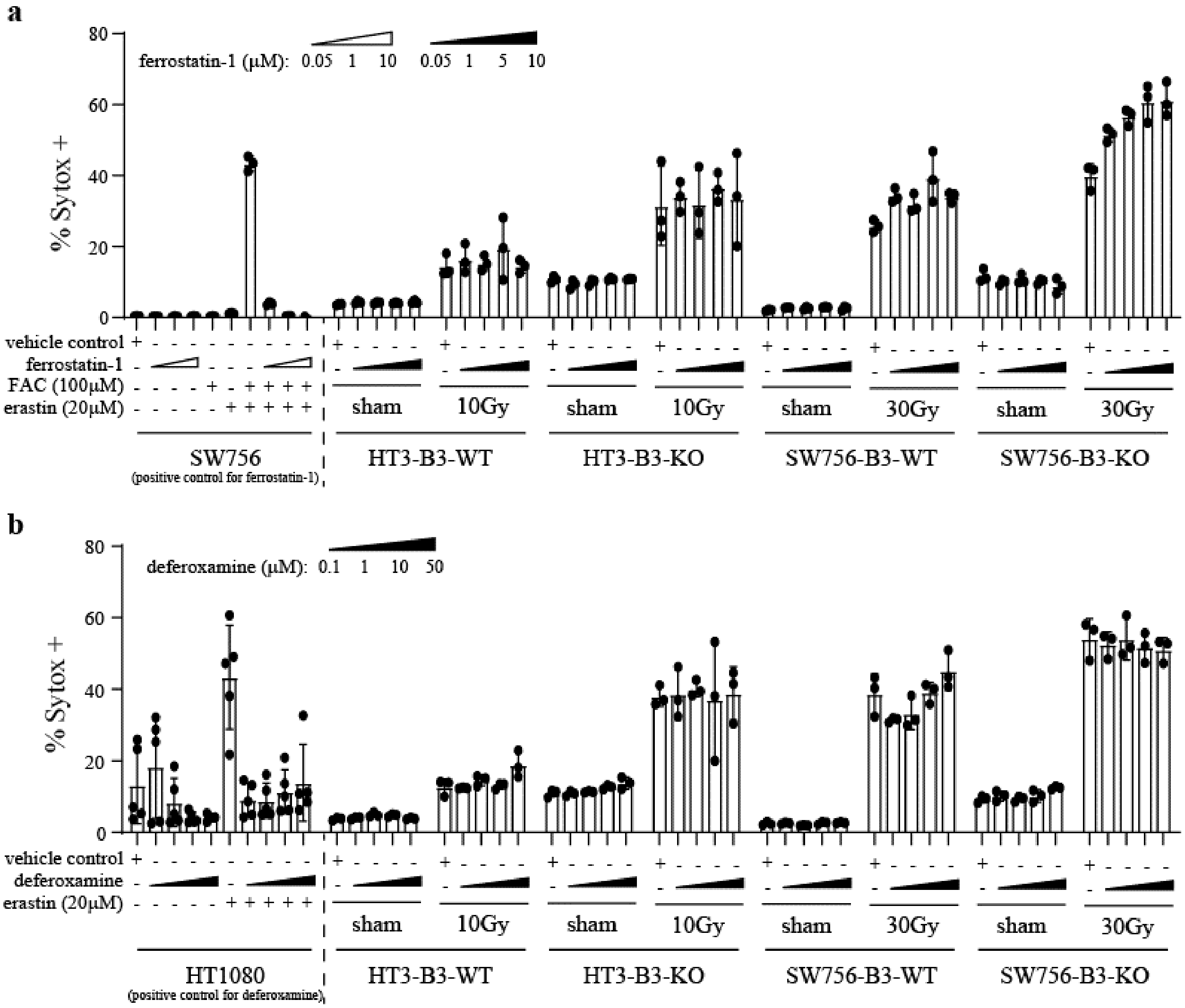

Supplemental Figure 4. a) Cell death (% Sytox +) induced by erastin (20μM) or radiation (10Gy for HT3, 30Gy for SW756) with or without increasing concentrations of ferrostatin-1. As a positive control for function of ferrostatin-1, SW756 parental cell line was treated with ferric ammonium citrate (FAC) to provide ample intracellular iron for one hour then treated with erastin and cell death measured by high content microscopy 17 hours later. Ferrostatin-1 was added 1 hour prior to erastin treatment where indicated. HT3-B3-WT and –B3-KO and SW756-B3-WT and –B3-KO cells were treated with sham or radiation followed by the addition of ferrostatin-1 24 hours later. Cell death was measured 96 hours after radiation. **b)** Cell death (% Sytox +) induced by erastin (20μM) or radiation (10Gy for HT3, 30Gy for SW756) with or without increasing concentrations of deferoxamine. As a positive control for function of deferoxamine, HT1080 cell line was treated with erastin and cell death measured by high content microscopy 17 hours later. Deferoxamine was added 1 hour prior to erastin treatment where indicated.

HT3-B3-WT and –B3-KO and SW756-B3-WT and –B3-KO cells were treated with sham or radiation followed by the addition of deferoxamine 72 hours later. Cell death was measured 96 hours after radiation. In both panels, two non-overlapping fields of view were imaged in each of n = 3-6 wells for each condition. The presented data is representative of one of three biologic replicates performed.

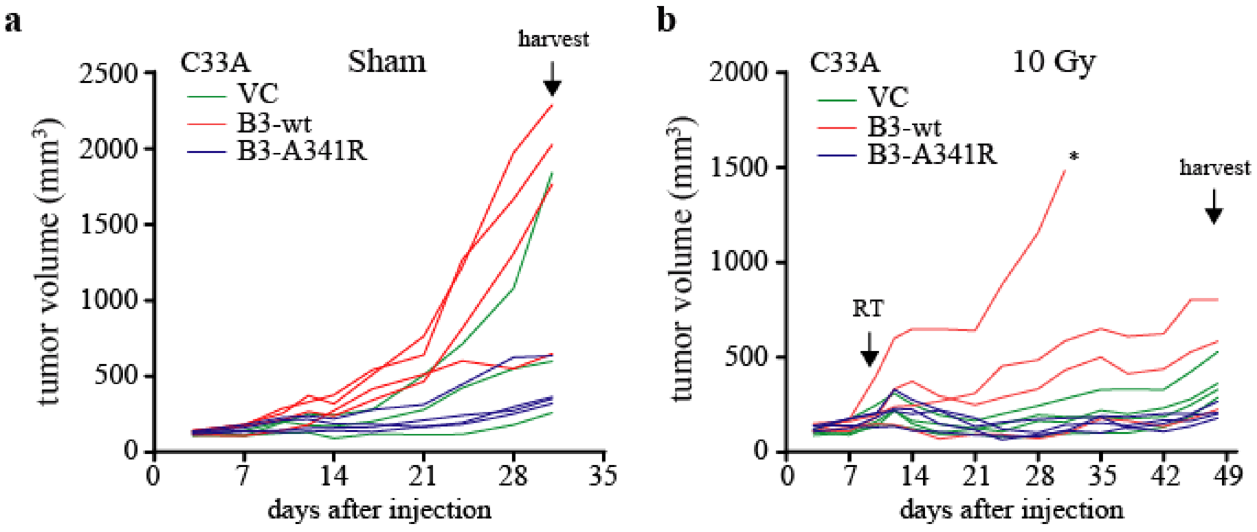

Supplemental Figure 5. Spider plots of absolute spheroid tumor volumes of C33A-VC, -B3, and – A341R tumors over time after injection following sham treatment (a) or 10Gy single fraction IR (b). Time of radiation is indicated by the arrow (RT). * = mouse harvested for ulcer formation. All other mice were sacrificed and tumors harvested at the indicated time point.

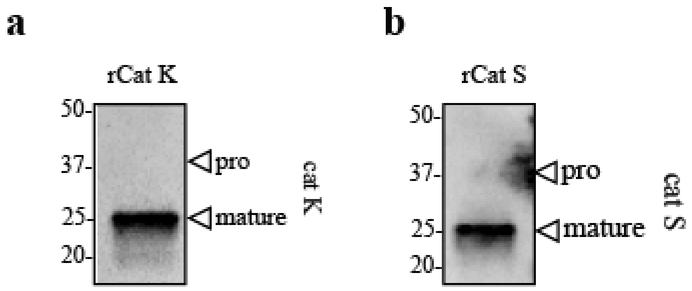

Supplemental Figure 6. Positive control for anti-cathepsin K and anti-cathepsin S antibodies. Western blot analysis of recombinant cathepsin K (a) and recombinant cathepsin S (b), which migrate at the size of the mature cathepsin form. These antibodies were used to probe lysates from SW756 cells (Figure 7i).

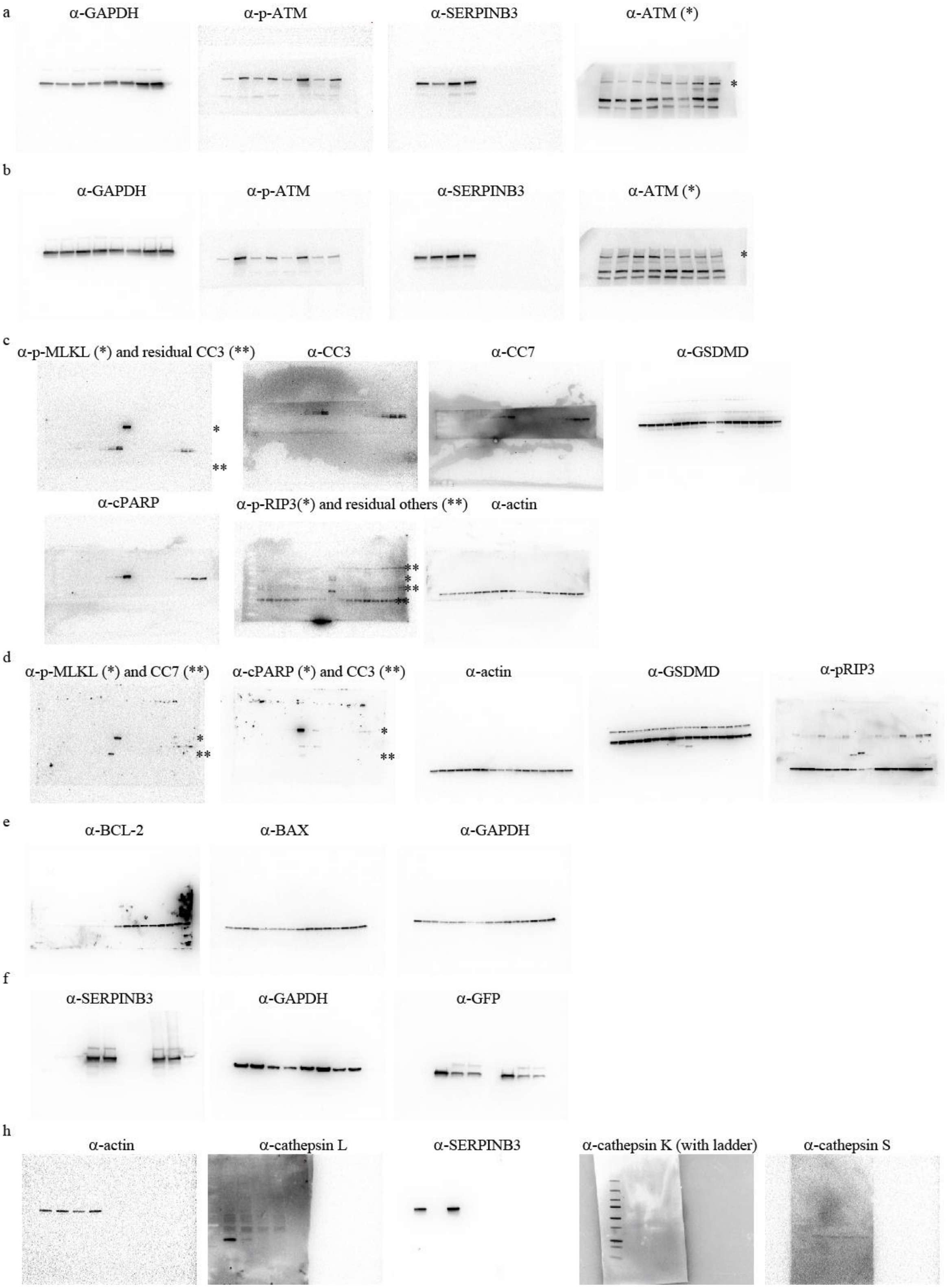

Supplemental Figure 7. Uncropped blots.

Uncropped blots for Figure 3g (a), Figure 3h (b), Figure 5a (c), Figure 5b (d), Figure 5e (e), Figure 7a (f), Figure 7i (g). Detected antigen is indicated above the blot.
