# Supplementary material for: SERPINB3 (SCCA1) inhibits cathepsin L and lysoptosis, protecting cervical cancer cells from chemoradiation
Source: Commun Biol. 2022 Jan 12;5:46. doi: 10.1038/s42003-021-02893-6 (PMC8755728; doi:10.1038/s42003-021-02893-6)
Supplement: Supplementary file 3 — Reporting Summary [file 42003_2021_2893_MOESM3_ESM.pdf]

nature research

Corresponding author(s): Stephanie Markovina

Last updated by author(s): October 28, 2021

Reporting Summary

Nature Research wishes to improve the reproducibility of the work that we publish. This form provides structure for consistency and transparency in reporting. For further information on Nature Research policies, see our Editorial Policies and the Editorial Policy Checklist.

Statistics

For all statistical analyses, confirm that the following items are present in the figure legend, table legend, main text, or Methods section.

n/a	Confirmed	
☐	☒	The exact sample size (*n*) for each experimental group/condition, given as a discrete number and unit of measurement
☐	☒	A statement on whether measurements were taken from distinct samples or whether the same sample was measured repeatedly
☐	☒	The statistical test(s) used AND whether they are one- or two-sided<br>*Only common tests should be described solely by name; describe more complex techniques in the Methods section.*
☒	☐	A description of all covariates tested
☐	☒	A description of any assumptions or corrections, such as tests of normality and adjustment for multiple comparisons
☐	☒	A full description of the statistical parameters including central tendency (e.g. means) or other basic estimates (e.g. regression coefficient) AND variation (e.g. standard deviation) or associated estimates of uncertainty (e.g. confidence intervals)
☐	☒	For null hypothesis testing, the test statistic (e.g. *F*, *t*, *r*) with confidence intervals, effect sizes, degrees of freedom and *P* value noted<br>*Give P values as exact values whenever suitable.*
☒	☐	For Bayesian analysis, information on the choice of priors and Markov chain Monte Carlo settings
☒	☐	For hierarchical and complex designs, identification of the appropriate level for tests and full reporting of outcomes
☒	☐	Estimates of effect sizes (e.g. Cohen's *d*, Pearson's *r*), indicating how they were calculated

Our web collection on statistics for biologists contains articles on many of the points above.

Software and code

Policy information about availability of computer code

Data collection	Gen5(TM) software from BioTek was used for high content imaging of cells in cell death experiments. Autodetection/object identification was optimized for most accurate counts (confirmed visually) for Sytox positive and Hoescht positive nuclei. Leica Application Suite X (LASX) was used to acquire live cell images.
Data analysis	ImageLab 5.2.1 was used to align, overlay and visualize Western blot images, Image J was used to determine densitometry when relevant. MatLab Prism 9 software was used to generate graphs and for statistical analysis of clonogenic survival assays, tumor doubling time, and survival curves. Volocity(R) software was used to quantitate LysotrackerDR/PI intensity per cell volume in Figure 6. QuPath was used to analyze IHC/TUNEL images in Figure 5 G-P.

For manuscripts utilizing custom algorithms or software that are central to the research but not yet described in published literature, software must be made available to editors and reviewers. We strongly encourage code deposition in a community repository (e.g. GitHub). See the Nature Research guidelines for submitting code & software for further information.

Data

Policy information about availability of data

All manuscripts must include a data availability statement. This statement should provide the following information, where applicable:

- Accession codes, unique identifiers, or web links for publicly available datasets
- A list of figures that have associated raw data
- A description of any restrictions on data availability

All raw data included in this manuscript including raw blot images, live cell image videos and TEM are available at: http://dx.doi.org/10.17632/8465mbnxt7.1, original slide images for TUNEL and CC3 staining of mouse tumors (Figure 5) are available at: https://app.histowiz.com/shared_orders/2ccb0ce8-e4e1-41d1-8f75-a0e621ced36e/slides/.

Field-specific reporting

Please select the one below that is the best fit for your research. If you are not sure, read the appropriate sections before making your selection.

☒ Life sciences ☐ Behavioural & social sciences ☐ Ecological, evolutionary & environmental sciences

For a reference copy of the document with all sections, see nature.com/documents/nr-reporting-summary-flat.pdf

Life sciences study design

All studies must disclose on these points even when the disclosure is negative.

Sample size	For in vivo mouse tumor model experiments, based on our published and preliminary in vitro data, using a Bonferroni correction with 0.05 significance, 80% power and a two-sided t-test, we estimated needing 4 mice per group to determine an effect size of 3.5 for tumor growth kinetics, and for irradiation experiments we expect a sample size of 3 mice per group is necessary to determine a very conservative effect size of 5 in tumor growth delay. Thus, 5 animals were innoculated with tumor allowing for 20% drop out. All animals that developed tumors were included in the analysis. For live cell time course imaging studies, the maximal number of fields were calculated as the number of fields that could be successfully imaged within the time points based on hardware limitations. For live-cell single time point imaging studies by confocal microscopy, and for gH2AX foci image quantitation, the sample size was n≥5 unless otherwise stated. Images presented are representative, and experiments to generate the images were repeated at least 3 times for biologic reproducibility. For 96 well plate imaging experiments n≥3 wells were used to measure intra-assay variability.
Data exclusions	For automated imaging assays, any wells/fields of view that were out-of-focus or otherwise immeasurable were excluded from data analysis. Mice that did not develop subcutaneous tumors were not included for randomzation in tumor growth/growth delay.
Replication	Cell-based experiments were replicated n≥3 times to ensure reproducibility
Randomization	Mice having developed tumors were randomized to sham or radiation groups. Cell based experiments were not randomized, but plated in replicates from the same stock plate/passage number.
Blinding	TEM images were scored by laboratory personnel blinded to the treatment and genotype.

Reporting for specific materials, systems and methods

We require information from authors about some types of materials, experimental systems and methods used in many studies. Here, indicate whether each material, system or method listed is relevant to your study. If you are not sure if a list item applies to your research, read the appropriate section before selecting a response.

Materials & experimental systems

n/a	Involved in the study	
☐	☒	Antibodies
☐	☒	Eukaryotic cell lines
☒	☐	Palaeontology and archaeology
☐	☒	Animals and other organisms
☒	☐	Human research participants
☒	☐	Clinical data
☒	☐	Dual use research of concern

Methods

n/a	Involved in the study	
☒	☐	ChIP-seq
☐	☒	Flow cytometry
☒	☐	MRI-based neuroimaging

Antibodies

Antibodies used	All antibody information including supplier, clone, dilution used and incubation parameters are described in the methods section.
Validation	Biologic positive and negative controls were used to validate the antibodies. Antibodies used were selected based on the published QA literature and confirmed in our hands as above.

Eukaryotic cell lines

Policy information about cell lines

Cell line source(s)	All parental ATCC lines were obtained directly from ATCC. Genetically modified lines were engineered from early passage parental cells.
Authentication	STR profiling was performed for any lines genetically modified and those passaged longer than 1 year from ATCC purchase.

Mycoplasma contamination

All cell lines were tested for mycoplasma every 3-6 months and any lines with evidence of mycoplasma infection were discarded and data since previous negative test were not used for anlysis.

Commonly misidentified lines
(See ICLAC register)

N/A

Animals and other organisms

Policy information about studies involving animals; ARRIVE guidelines recommended for reporting animal research

Laboratory animals	Mouse tumor studies used female athymic nude mice obtained from Charles River Labs CRL:NU (NCR)-FOXN1 NU (Homozygous), injected at 6-8 weeks of age. Only female mice were utilized given the hormonal/genetic background of patients with cervical cancer which occurs exclusively in women.
Wild animals	N/A
Field-collected samples	N/A
Ethics oversight	The methods were performed in accordance with relevant guidelines and regulations and approved by the Washington University Institutional Biological & Chemical Safety Committee. All mouse experiments were performed in accordance with relevant guidelines and regulations and approved by the Washington University Institute Institutional Animal Care and Use Committee (Protocol #20-0470).

Note that full information on the approval of the study protocol must also be provided in the manuscript.

Flow Cytometry

Plots

Confirm that:

☒ The axis labels state the marker and fluorochrome used (e.g. CD4-FITC).

☐ The axis scales are clearly visible. Include numbers along axes only for bottom left plot of group (a 'group' is an analysis of identical markers).

☐ All plots are contour plots with outliers or pseudocolor plots.

☐ A numerical value for number of cells or percentage (with statistics) is provided.

Methodology

Sample preparation	Cells were cultured under indicated conditions for the indicated time then trypsinized (combined with non-adherent cells), permeabilized with a Triton-X citrate buffer containing propidium iodide, and analyzed by flow cytometer immediately after 40 minute incubation and filtration through a mesh strainer
Instrument	MACSQuant Analyzer 10
Software	FlowJo(TM) v10.7
Cell population abundance	Single cells comprised ~90% of the total events.
Gating strategy	Cells were gated by excluding debris on FSC-A (X axis) v SSC-A (y-axis) the single cells were gated on FSC-A vs FSC-H. This population was subject to cell cycle analysis on PI-A histogram for cell count (y-axis). Raw data are included in the revised manuscript supplemental.

☒ Tick this box to confirm that a figure exemplifying the gating strategy is provided in the Supplementary Information.
